# UCP2 and UCP3 polymorphisms as risk factors of insulin resistance for Indonesian obese adolescents

**DOI:** 10.1186/1687-9856-2013-S1-P105

**Published:** 2013-10-03

**Authors:** M Mexitalia, Agustini Utari, Suci Romadhona, Taro Yamauchi, Takafumi Ishida

**Affiliations:** 1 Department of Pediatrics, Faculty of Medicine, Diponegoro University/ Dr.Kariadi Hospital, Semarang, Indonesia; 2 Laboratory of Human Ecology, Graduate School of Health Sciences, Hokkaido University, Sapporo, Japan; 3 Laboratory of Human Biology and Genetic, Department of Biological Sciences, Graduate School of Sciences, University of Tokyo, Tokyo, Japan

## 

Uncoupling Protein 2 (UCP2) dan Uncoupling Protein 3 (UCP3) is a mitochondrial transmembrane carriers which uncouple the transport of protons across the inner mitochondrial membrane from electron transport and the synthesis of ATP from ADP. The UCPs play a role in energy homeostasis and have considered to be candidate gene for controlling obesity and insulin resistance. The aim of our study is to analyze UCP2 and UCP3 polymorphisms as risk factors of insulin resistance in obese adoslescent.

A case control study was conducted in Junior High School in Semarang, Central Java, Indonesia during 2007- 2008. Seventy five subjects included in this study. Height, weight and body mass index (BMI) were measured. Insulin resistance was estimated by using the homeostasis model assessment (HOMA-IR) score which calculated as fasting insulin (microU/mL) x fasting glucose (mmol/L)/22.5. Three polymorphic sites i.e. UCP3 -55c/t, UCP3 Y210Y, and UCP2 A55V were investigated by using the Restriction Length Fragment Polymorphism. The data were analyzed by 2x2 table and odd rasio with 95% confidence interval.

There were 38 obese (27 male, 11 female) and 37 normoweight ( 25 male, 12 female) subject with the mean age was 13.2 (SD 0.32) years. Of 38 obese, there were 3 (7.9%) subject had insulin resistance. None of normoweight subjects had insulin resistance. There was a significant correlation between BMI and insulin resistance (r = 0. 601, p< 0.001). Of 38 obese subject, C allele of UCP2 A55V and C allele of UCP3 Y210Y are a risk factors of insulin resistance (OR 2.45, 95% CI 1.97-3.04 , p< 0.0001 ; OR 2.6, 95% CI 2.10-3.24, p< 0.0001 respectively), whereas UCP3 -55c/t was not considered as risk factors of insulin resistance.

Our study suggest that C allele of UCP2 A55V and C allele in UCP3 Y210Y are a risk factors of insulin resistance in Indonesian obese adolescent. Larger studies is needed to prove the role of UCPs in controlling insulin resistance.

